# Abdominal heterotopic pregnancy after *in vitro* fertilization and embryo transfer following bilateral salpingectomy: A case report and literature review

**DOI:** 10.3389/frph.2022.921141

**Published:** 2022-07-18

**Authors:** Yifeng Liu, Yu Li, Keting Li, Shuangdi Li

**Affiliations:** ^1^Department of Gynecology, Shanghai First Maternity and Infant Hospital, Tongji University School of Medicine, Shanghai, China; ^2^Department of Ultrasonography, Shanghai First Maternity and Infant Hospital, Tongji University School of Medicine, Shanghai, China

**Keywords:** assisted reproductive technology, bilateral salpingectomy, heterotopic pregnancy, intraperitoneal hemorrhage, laparoscopy

## Abstract

**Background:**

Risk of heterotopic pregnancy following bilateral salpingectomy has increased considerably due to the widespread use of assisted reproductive technology. Poor understanding of this condition often causes delayed or missed diagnosis.

**Objective:**

In this report, we describe the case of a 30-year-old pregnant woman with lower abdominal pain lasting for half a day and a history of bilateral salpingectomy. Two embryos had been transferred 21 days preceding her presentation.

**Methods:**

Case report.

**Results:**

Laparoscopic surgery revealed intraperitoneal hemorrhage and proper ovarian ligament pregnancy confirmed by histopathology. Viable intrauterine pregnancy was verified 3 days later by ultrasound examination.

**Conclusion:**

Heterotopic pregnancy is a serious condition that may be life-threatening. Clinicians should be aware of the potential for heterotopic pregnancy in patients receiving *in vitro* fertilization and embryo transfer after bilateral salpingectomy.

## Introduction

Bilateral salpingectomy (BS) is often performed as a surgical treatment for repeated ectopic pregnancy (EP) or bilateral hydrosalpinx. Although rare, EP can still occur in these patients following *in vitro* fertilization and embryo transfer (IVF-ET). Nearly 20 ectopic pregnancies have been reported after IVF-ET in patients with previous BS (1, 2). This occurrence presents a clinical conundrum for gynecologists as well as unnecessary disputes between doctors and patients.

Heterotopic pregnancy (HP) is defined as simultaneous intrauterine and extrauterine pregnancy (3). The incidence of HP has increased from 0.03 to 1–3% due to the widespread use of assisted reproductive techniques (4). In HP, EP mostly occurs in the ampulla or interstitial part of the fallopian tube (5). However, in patients with previous BS, despite the removal of both tubes, HP can still occur in the tube stump or the abdominal cavity (6). In these patients, HP can be easily misdiagnosed as a singular intrauterine pregnancy and can cause life-threatening outcomes. Here, we present a case of abdominal HP following IVF-ET after BS and provide a comprehensive review of the literature. Written informed consent for publication of this report and accompanying images was obtained from the patient.

## Case description

**Figure 1:**
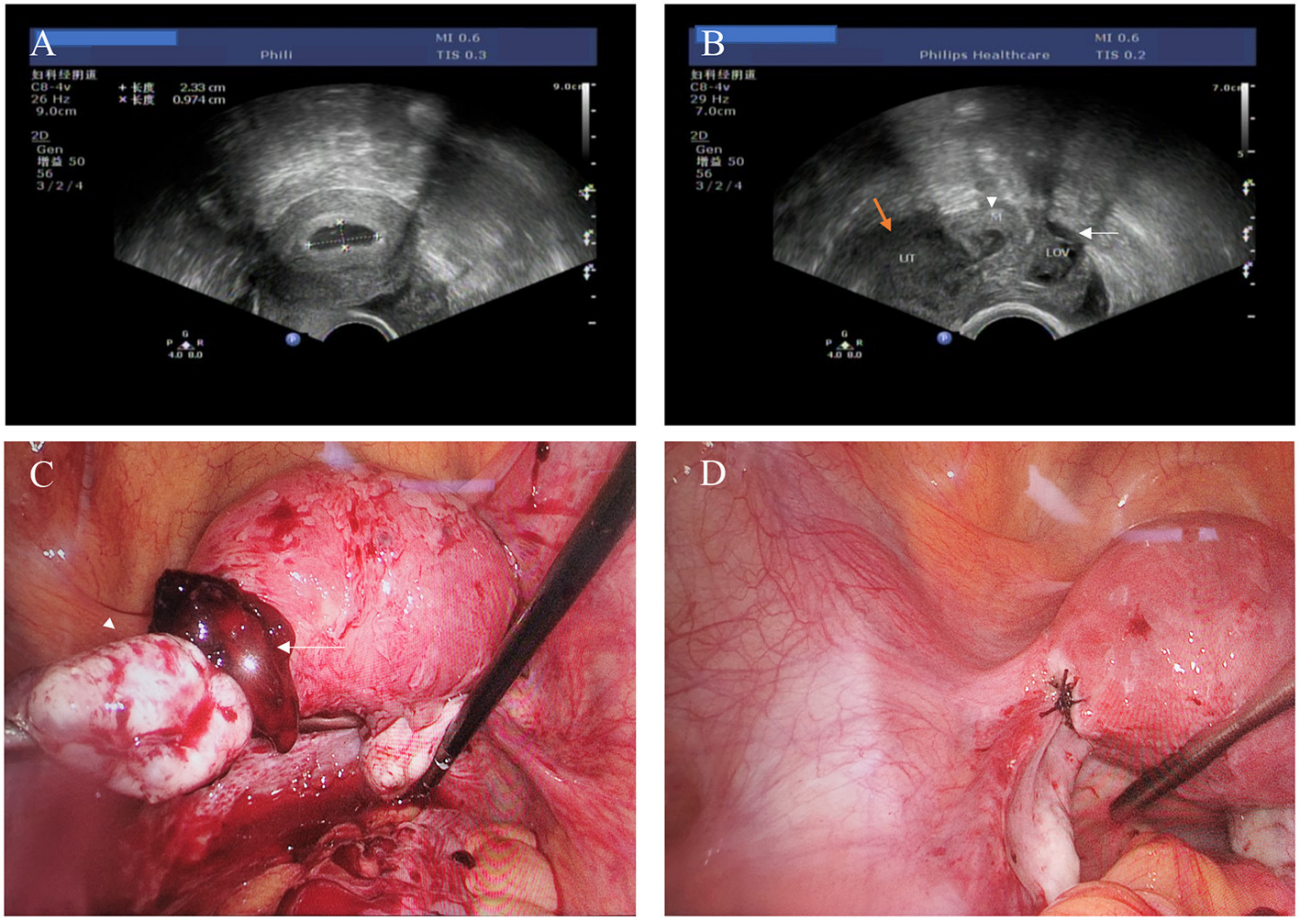
**(A)** Transvaginal ultrasound examination showing an intrauterine gestational sac. **(B)** Transvaginal ultrasound examination showing a hypoechoic mass (white arrowhead) between the uterus (orange arrow) and left ovary (white arrow). **(C)** Laparoscopic findings of an approximately 2-cm blood clot containing chorionic tissues attached to the left ovarian proper ligament. **(D)** Suture repair of the active bleeding point on the surface of the ligament.

A 30-year-old pregnant woman (gravida 3, para 0) presented at our gynecological emergency department for lower abdominal pain lasting for half a day accompanied by nausea and vomiting twice. She had undergone IVF-ET with two transferred embryos 21 days preceding this presentation. Transvaginal Color Doppler ultrasound examination revealed an intrauterine gestational sac measuring 23 × 10 × 22 mm along with a mixed mass measuring 28 × 24 × 20 mm in the left adnexal area with blood signals in the mass. An intrapelvic hypoechoic area of approximately 71 × 29 × 29 mm suggested blood clot formation (Figure 1). The patient had tenderness in her left abdomen but was hemodynamically stable. Her hemoglobin level was 140 g/L at admission and her serum beta-hCG levels were 2,607.20 mIU/mL and 8,404.10 mIU/mL 48 h apart.

The patient had a history of polycystic ovary syndrome and had been infertile for 10 years. She had no history of autoimmune disease or other comorbidities. In 2014, she attempted IVF-ET and had undergone laparoscopic right salpingectomy due to a right tubal pregnancy. In February 2021, she had been diagnosed with a left ovarian pregnancy 32 days after IVF-ET. A left partial ovarian resection was performed to remove the pregnancy tissues, and the left fallopian tube was removed at the patient's request.

During this visit, the patient underwent immediate laparoscopic surgery. The intra-abdominal hemorrhage volume was approximately 700 mL. A blood clot measuring approximately 2 cm was attached to the left proper ovarian ligament, in which chorionic tissues with a diameter of ~0.5 cm were observed. The surface of the ligament bled actively after the clot was removed and was repaired using a zigzag suture. Both tube stumps appeared to be normal. The patient was discharged in good health on day 4 after the surgery. Histopathological examination confirmed an abdominal pregnancy. The patient then underwent another transvaginal ultrasound examination 7 days after the surgery, which revealed a viable intrauterine pregnancy of about 45 days, with fetal pole and a beating heart (Figure 1). Finally, the woman went through an uncomplicated pregnancy which resulted in the delivery of a full term (41^+3^ weeks) healthy female neonate weighting 3350g by Cesarean Section, due to woman's desire.

## Methods and search strategy

A comprehensive review of the literature was conducted. Inclusion criteria for full manuscription evaluation were studies that reported case(s) or case series of HP following IVF-ET after previous BS. A search of the PubMed database for English literature published up until September 10, 2021, was performed by using the terms “(bilateral salpingectomy) AND (ectopic pregnancy) OR (Heterotopic Pregnancy)”. Data was extracted by the author Y. L. and double-checked by the author S.L.

## Discussion

This study presented a case of abdominal HP following IVF-ET after BS. Additionally, through literature search, altogether 15 articles reporting 20 cases were found, including one patient with recurrent HPs, which was counted as a single case (7). The details of all 20 cases are listed in Table 1.

**Table 1:** Clinical characteristics of HPs after IVF-ET in patients with previous bilateral BS.

**No**.	**Reference**	**Age (years)**	**Indication for bilateral salpingectomy**	**Number of transplanted embryos**	**Symptoms**	**Bleeding volume**	**EP site**	**Treatment**	**Intrauterine pregnancy outcome**
1	(26)	27	Bilateral hydrosalpinx	2	Severe abdominal pain	Transfused with 2 units of blood	Right uterine cornua	Laparoscopic right uterine cornual pregnancy resection	Gestational to full term, cesarean section
2	(8)	28	Bilateral salpingitis	3	Severe abdominal pain	1,400 mL of free intraperitoneal blood	Abdominal cavity (posterior uterine wall)	Laparoscopic resection of the abdominal pregnancy	Gestation to 38 weeks, cesarean section
3	(7)	35	Two IVF failures	2 (third IVF cycle) 2 (fourth IVF cycle)	Asymptomatic	A large amount of fluid in the pouch of Douglas (third cycle) NA (fourth cycle)	Right fallopian tube stump (third cycle) Left fallopian tube stump (fourth cycle)	Laparoscopic right and left fallopian tube stump resection	Missed and spontaneous abortion
4	(27)	31	Left hydrosalpinx, right ovarian cyst	2	Abdominal pain	2,000 mL of intraperitoneal blood	Left fallopian tube stump	Laparotomy for left cornuectomy	Gestation to 37 weeks, cesarean section
5	(28)	35	Unilateral tubal pregnancy + contralateral fallopian tube anatomical destruction	3	Cramping lower abdominal pain	Transfused with 2 units of blood	Right fallopian tube stump	Laparoscopic right fallopian tube stump resection	Gestation to full term, cesarean section
6	(10)	30	Bilateral tubal pregnancy	4	Asymptomatic	NA	Abdominal cavity (the omentum)	Laparotomy for partial omentectomy	Gestation to 36 weeks, cesarean section
7	(29)	31	Bilateral tubal pregnancy	3	Painless vaginal spotting	NA	Left fallopian tube stump (interstitial, twin pregnancy)	Laparotomy for left cornuectomy	Twin pregnancy, gestation to 38 weeks, cesarean section
8	(30)	37	Bilateral hydrosalpinx	4	Abdominal pain	Transfused with 2 units of blood	Left fallopian tube stump (interstitial)	Laparotomy for left cornuectomy	Abortion 5 weeks after surgery (Trisomy 21)
9	(6)	28	2 right tubal pregnancies + left hydrosalpinx	2	Lower abdominal pain	Approximately 2,000 mL of hemoperitoneum	Right ovary	Laparoscopic resection of right ovarian pregnancy	Singleton term pregnancy
10	(31)	30	Bilateral ectopic pregnancies	3	Lower abdominal pain and vaginal bleeding	NA	Right uterine cornua	Laparotomy for right cornuectomy	Abortion
11	(32)	38	Bilateral ectopic pregnancies	4	Lower abdominal pain and peritoneal irritation	Approximately 500 mL of hemoperitoneum	Left uterine cornua	Laparotomy for left cornuectomy	Twin pregnancy, gestation to 36 weeks, cesarean section
12	(12)	33	Bilateral ectopic pregnancies	2	Pain in the left lumbar back after curettage	NA	Between the retroperitoneal retrorenal fascia and left psoas major muscle	Laparotomy and 10 mg of methotrexate	Embryo arrest
13	(11)	38	Tubal blockage	2	Abdominal pain	Approximately 1,200 mL of hemoperitoneum	Left upper abdominal quadrant near the spleen	Laparotomy and cesarean section	Twin pregnancy, gestation to 26 weeks, cesarean section
14	(33)	29	HP with failed intrauterine pregnancy	2	A sudden and worsening pelvic pain	Approximately 700 mL of hemoperitoneum	Left uterine cornua	Laparoscopic left cornual resection	Gestation to 39 weeks, cesarean section
15	(9)	NA	Unknown	Unknown	Vaginal bleeding for 11 days	NA	Right interstitial pregnancy	Local methotrexate (30 mg)	NA
16	(9)	NA	Bilateral ectopic pregnancies	3	Asymptomatic (found in routine ultrasound)	NA	Left interstitial pregnancy	Suction and local methotrexate (20 mg) followed by cornual resection 2 weeks later for mass eruption	NA
17	(9)	NA	Bilateral ectopic pregnancies	3	Abdominal pain for 3 days	1,400 mL of abdominal hematocele	Posterior uterine wall	Laparotomy mass removal	NA
18	(9)	NA	Unknown	2	Vaginal bleeding and abdominal distension for 6 days	2,000 mL of abdominal hematocele	Right cornual	Laparotomy cornual removal	NA
19	(9)	NA	Unknown	2	Asymptomatic (found in routine ultrasound)	NA	Right cornual	Laparotomy cornual resection	NA
20	Current	30	Bilateral ectopic pregnancies	2	Abdominal pain for half a day	Approximately 700 mL of hemoperitoneum	Right proper ovarian ligament	Laparoscopic mass removal	Gestation to 41 weeks, cesarean section

*HP, heterotopic pregnancy; IVF-ET, in vitro fertilization and embryo transfer; BS, bilateral salpingectomy; EP, ectopic pregnancy NA, not available or not applicable*.

These HPs could be grossly classified into three categories according to EP site: (1) embryos implanted at the tubal stump/interstitial part/cornua uteri, which accounted for most cases (14/21, 66.7%); (2) embryos implanted in the abdominal cavity including the posterior uterine wall (8, 9), the omentum (10), near the spleen (11), the ovary, (6) and the proper ovarian ligament (as in the current case), altogether accounting for 28.6% of cases (6/21); and (3) embryos implanted in the retroperitoneum, which included one case (1/21, 4.7%) (12).

The reason for the occurrence of EP after BS remains unclear. Since the tube stump or the interstitial/cornual section is directly connected to the uterine cavity, the mechanism of these EPs may be the abnormal migration and implantation of the transplanted embryos into the uterus; however, it is counterintuitive that EP occurs in the extrauterine area following BS. Transfered embryos may migrate through the fistula formed at the uterine corner following resection of the fallopian tube and implant in the abdominal cavity. One possible explanation for retroperitoneal EP is the transport of the embryos to the implantation site by the lymphatic channel, although no direct evidence supports this hypothesis (13).

The widespread application of IVF-ET has greatly increased the risk for HP, even after BS. For example, fifty HPs were reported in a single center over 8 years, including five cases with previous BS (9). It should be noted that all the patients included in our review had received multiple embryos transfer (Table 1). We thought our results highlighted the importance of elective single-embryo transfer (eSET) policy in clinical practice (14). eSET can greatly reduce the possibility of multiple pregnancies and therefore the possibility of HP. However, it has also been reported that a patient who had developed right tubal pregnancy and omental/peritoneal trophoblastic heterotopic pregnancy after received single embryo transfer (15). But this situation is extremely rare, and the cause of HP is considered as implantation of trophoblastic tissues on the omentum/peritoneum rather than embryo splitting (15).

Our case was unique in that HP developed at the left proper ligament of the ovary, and the patient's previous EP had occurred in the left ovary; thus, there seemed to be a predisposition in this patient for the embryo to be transported to the left adnexal area. Embryonic implantation is similar to leukocyte migration, with steps of apposition, attachment, and implantation. As leukocyte migration is highly chemotactic, we have previously speculated that EP may develop from competition between the signals derived from the endometrium and the fallopian tube (16). In this case, we hypothesized that abnormal cytokines or chemokines from the left ovary caused the blastocysts to migrate through a microfistula in the left corner of the uterus, followed by implantation on the ovary or at the proper ovarian ligament.

It has been reported that inflammatory diseases or autoimmune diseases may increase the rate of EP. For instance, the odds of EP significantly increased in Crohn's disease as compared to inflammatory bowel diseases free controls (17). The incidence of HP may also rise in patients with these comorbidities. However, for all the patients included in our review, no medical histories for such diseases were mentioned in the original papers. More attention should be paid to the relationship between systematic diseases and the onset of HP.

A major concern in HP is that as the intrauterine pregnancy continues to grow, the risk of uterine rupture may increase, especially in patients with HP who have undergone tube stump resection or cornuectomy. In addition, the possibility of a gradual enlargement of a microfistula in the corner of the uterus to dehiscence cannot be ruled out. In fact, more than 40 cases of spontaneous uterine rupture during pregnancy following salpingectomy have been reported until 2018 (18). In our report, none of the HP cases reviewed involved uterine rupture, despite the fact that some patients had undergone cornuectomy for the treatment of simultaneous cornual pregnancy. This result may have been related to the high vigilance of doctors and close monitoring during pregnancy.

The most important aspect of HP following BS is that many patients have massive intraperitoneal hemorrhage at diagnosis. In the literature, 14 patients (14/21, 66.7%) had hemoperitoneum exceeding 500 mL or required a blood transfusion, while six patients (6/21, 28.6%) had internal bleeding >1,000 mL. There may be several reasons for this. First, many doctors are not aware that EP can occur after BS; thus, the diagnosis is often delayed or even missed. Second, simultaneous intrauterine pregnancy can act as a confounding factor. The symptoms of HP are usually not obvious, as vaginal bleeding is not common in these patients. Third, the patient may be assured of their intrauterine pregnancy and neglect minor discomforts that may be signs of EP; thus, they often present with sudden and severe abdominal pain (Table 1).

HP is a high-risk situation. The most effective diagnostic method is transvaginal sonography. Careful transvaginal ultrasound evaluation should be performed in any patients suspicious of HP with or without BS, especially when patients who have received multiple embryos transfer. Color Doppler imaging showed little improvement for the identification of EP (19, 20). A recently described novel sonographic sign, “the double corpus luteum”, may be a characteristic sign for spontaneous HP but not suitable for HP patients after IVF-ET (21). Magnetic resonance imaging may improve the diagnostic accuracy of HP by detecting abdominal pregnancy more sensitively. However, its usage in HP is still controversial, as there are not enough studies proving the safety of MRI in the first trimester (22). Series serum hCG concentration measurement is of little clinical significance as the co-existed intrauterine pregnancy may confound the rise of hCG levels.

The treatment of HP includes expectant therapy, medical therapy, and surgery. Surgery is considered as the first choice of treatment. All the patients reviewed in this report had received surgery except for one patient who had taken local methotrexate (MTX) injection (9). The outcome of the intrauterine pregnancy for this patient was not mentioned in the original report (9). Although a recent study has showed that MTX administration can be safe and effective in treating cervical heterotopic pregnancy with successful perinatal outcome (23), it is still contraindicated when the continuation of intrauterine pregnancy is desired. Compared to MTX therapy, surgery can treat HP effectively and timely. However, the effects of surgery on the perinatal outcome are still controversial. Miscarriage rates reached up to 26% in women who had surgical treatment (24). Others demonstrated that surgical treatment of heterotopic extrauterine pregnancies did not increase the risk of miscarriage of the concomitant live eutopic pregnancy (25). In our report, among 14 patients who received surgery with pregnancy outcomes, 10 patients resulted in the delivery of full-term neonates, while 2 patients had spontaneous abortion or fetal arrest. Although 66.7% (10/15) patients had satisfactory pregnancy outcomes, the sample size is too sample to draw a definitive conclusion. Future cohort trials are needed to elucidate the impact of surgery on the pregnancy outcome.

The strength of our report is that we reported a rare case of HP after IVF-ET following BS. Assisted reproduction is in greater demands at present time and the incidence of HP increased dramatically due to the wide use of assisted reproduction technology. Our report highlighted the fact that clinicians should be aware of the potential for HP in patients receiving IVF-ET after BS, especially in those who have received multiple embryos transfer. Close ultrasound examination should be performed at the time of initial diagnosis and follow-up visits should be scheduled in high-risk women to exclude this diagnosis. The limitation of our report is it only include one patient and more cases are needed to clarify the risk factors, diagnostic methods, practice points of HP after IVF-ET.

## Data availability statement

The original contributions presented in the study are included in the article/supplementary material, further inquiries can be directed to the corresponding author/s.

## Ethics statement

Ethical approval was obtained from the Ethics Committee of Tongji University Shanghai First Maternity and Infant Hospital (Reference No. KS21293). Written informed consent for participation in and the publication of this case report and accompanying images was obtained from the patient.

## Author contributions

YLiu interpreted the patient data and wrote the first draft of the article. YLi collaborated in the writing drafts. KLi provided the photos of transvaginal ultrasound examination and interpreted the patient data. SLi collected materials, interpreted the patient data, and revised the article. All authors have read and approved the manuscript.

## Funding

The study was supported by grants awarded to SLi by Science and Technology Commission of Shanghai Municipality (No 21Y11907700). The founding organization has no part in the collection, analysis and interpretation of data and in writing the manuscript.

## Conflict of interest

The authors declare that the research was conducted in the absence of any commercial or financial relationships that could be construed as a potential conflict of interest.

## Publisher's note

All claims expressed in this article are solely those of the authors and do not necessarily represent those of their affiliated organizations, or those of the publisher, the editors and the reviewers. Any product that may be evaluated in this article, or claim that may be made by its manufacturer, is not guaranteed or endorsed by the publisher.

## Abbreviations

HP: Heterotopic pregnancy
Hcg: human chorionic gonadotropin
IVF-ET: *In vitro* fertilization-embryo transfer
BS: bilateral salpingectomy.
